# Soap is not enough: handwashing practices and knowledge in refugee camps, Maban County, South Sudan

**DOI:** 10.1186/s13031-015-0065-2

**Published:** 2015-12-20

**Authors:** Raina M Phillips, Jelena Vujcic, Andrew Boscoe, Thomas Handzel, Mark Aninyasi, Susan T Cookson, Curtis Blanton, Lauren S Blum, Pavani K Ram

**Affiliations:** Centers for Disease Control and Prevention, Atlanta, Georgia; University of Buffalo, Buffalo, NY USA; Oxfam International, Juba, South Sudan; Dakar, Senegal

**Keywords:** Handwashing, Hygiene, Sanitation, Soap, Emergencies, Refugees, South Sudan, Structured observation

## Abstract

**Background:**

Refugees are at high risk for communicable diseases due to overcrowding and poor water, sanitation, and hygiene conditions. Handwashing with soap removes pathogens from hands and reduces disease risk. A hepatitis E outbreak in the refugee camps of Maban County, South Sudan in 2012 prompted increased hygiene promotion and improved provision of soap, handwashing stations, and latrines. We conducted a study 1 year after the outbreak to assess the knowledge, attitudes, and practices of the refugees in Maban County.

**Methods:**

We conducted a cross sectional survey of female heads of households in three refugee camps in Maban County. We performed structured observations on a subset of households to directly observe their handwashing practices at times of possible pathogen transmission.

**Results:**

Of the 600 households interviewed, nearly all had soap available and 91 % reported water was available “always” or “sometimes”. Exposure to handwashing promotion was reported by 85 % of the respondents. Rinsing hands with water alone was more commonly observed than handwashing with soap at critical handwashing times including “before eating” (80 % rinsing vs. 7 % washing with soap) and “before preparing/cooking food” (72.3 % vs 23 %). After toilet use, 46 % were observed to wash hands with soap and an additional 38 % rinsed with water alone.

**Conclusions:**

Despite intensive messaging regarding handwashing with soap and access to soap and water, rinsing hands with water alone rather than washing hands with soap remains more common among the refugees in Maban County. This practice puts them at continued risk for communicable disease transmission. Qualitative research into local beliefs and more effective messaging may help future programs tailor handwashing interventions.

## Background

Overcrowded conditions, poor nutrition, and limited water, sanitation, and hygiene (WASH) facilities put refugees at high risk for communicable disease outbreaks, especially diseases transmitted by the fecal-oral route such as diarrhea and viral hepatitis A and E. In one meta-analysis, handwashing with soap has been shown to reduce diarrhea risk by 31 % and acute respiratory infection risk by 21 % [1]. Washing with soap is more effective at hand decontamination than washing with water alone [2–4].

Studies on handwashing frequency, motivators, and barriers have primarily been performed in stable developing country contexts [5]. Curtis et al. performed an eleven country study and found in structured observations that 17 % of caregivers washed hands with soap after defecation, while 45 % rinsed hands with water alone [5]. However, in the setting of internal displacement or among refugees, habit and cultural norms can be disrupted, thereby potentially altering practices such as handwashing. There is a dearth of information on handwashing frequency, motivators and barriers in refugee settings. A study in three refugee camps by Biran et al. (in Kenya, Ethiopia and Thailand) found that soap was used for handwashing after 20 % of toilet use events [6]. In qualitative research, they found that barriers to handwashing included lack of free soap and preference to use soap for laundry. They concluded that handwashing rates were suboptimal despite hygiene education in the camps [6]. A study in a Malawian refugee camp on soap presence and diarrhea found the presence of soap in households to be protective against diarrhea, suggesting the soap was being used for handwashing, however structured observation was not performed to verify this practice [7].

Maban County, Upper Nile State, South Sudan experienced an influx of refugees escaping violence and civil unrest in Sudan beginning in November 2011. As of December 2013, approximately 124,000 registered refugees lived in four camps in Maban County: Kaya, Yusuf Batil, Doro, and Gendrassa [8, 9]. In 2012, a large outbreak of hepatitis E virus (HEV) occurred among refugees in all four of the camps. Approximately 11,000 persons were symptomatic with HEV and, among them, 238 deaths occurred [8]. In response to the HEV outbreak, non-governmental organizations (NGOs) increased hygiene promotion efforts including messaging about handwashing, soap use, and HEV transmission.

The four camps varied in size and demographics. Doro was predominantly Christian while Kaya, Batil, and Gendrassa were predominantly Muslim. The hepatitis E outbreak disproportionately affected individuals in Batil and what is now Kaya camp due to their physical locations in a floodplain. Different organizations were in charge of WASH activities for each camp and, thus, some camps had more handwashing stations built, others had better family:latrine ratios, and clean water provision was a priority in all camps. Latrines and, if present, handwashing stations, were shared among multiple compounds of families in each camp. The hygiene promotion also varied from camp to camp in response to the outbreak. While all agencies focused on improving access to clean water, other interventions included posters depicting important times to wash hands with soap, community health workers who provided family and school level messaging regarding handwashing, and increasing latrine and handwashing station access. The amount of soap distributed to each household monthly increased equally amongst all camps.

In this study, we sought to describe the knowledge, attitudes, and practices among the refugees in three camps in Maban County 1 year after the scale up of hygiene education, increased soap distribution, and improved sanitation measures in response to the HEV outbreak. We also sought to examine the motivators and barriers to handwashing with soap including access to latrines and soap and beliefs about disease risk and transmission. Finally, we aimed to compare self-reported practices with the observed rates of handwashing with soap and rinsing with water at critical times for pathogen transmission to assess the validity of self-reported handwashing behavior compared to directly observed behavior.

## Methods

### Selection and enrollment of respondents

From November 22 through December 19, 2013, during the dry season, we conducted cross-sectional surveys of women over 18 years in each of three refugee camps (Kaya, Yusuf Batil, and Doro). Households were selected by systematic random sampling from the UNHCR list of registered refugees of each camp. We aimed for a sample size of 200 per camp based on the following assumptions: 50 % of households would have bar soap and water at the handwashing station near the latrine; precision based on 95 % confidence interval of +/− 7 %. We increased the sample size to 230 households for Kaya and Batil and to 250 for Doro to account for non-response rate seen in the prior two camps.

Eligible respondents were adult females, preferably female heads of household; if an adult female member of the household was not present, we returned at least one additional time to attempt to identify an eligible respondent. If still not present, another household on the sample list was chosen. Due to low literacy levels, verbal informed consent was obtained from all participants after an explanation of the study objectives. Participants were informed of their right to refuse to participate or answer individual questions.

We developed a standardized questionnaire in English and translated it into Arabic with the enumerators. The questionnaire was piloted in Arabic and changes were made based on field testing. We collected information about demographics, handwashing knowledge, practices, and preferences, and HEV and diarrhea transmission knowledge. Questions regarding important times to use soap and when the respondent used soap were asked in both open-ended and closed-ended formats. Availability of soap and water was directly observed at latrine handwashing stations and in the household compound.

We sought to conduct structured observation among a subset of 50 households per camp identified by systematic random sampling from the selected sample list of households from the UNHCR list. We felt structured observation was a critical complement to the survey as self-reported practices are known to be biased. A trained data collector observed the routine household activities and handwashing events for three hours, aiming to capture handwashing behavior–none, water alone, or soap and water– during critical events, such as before preparing or cooking food, before eating, before feeding a child, after toileting, after cleaning a child’s bottom, and after disposing a child’s feces using methods as previously described [10]. The ages of the household individuals was approximated by the observer as 15 years and younger or over 15 years of age. Although we preferred women to be observers for any cultural considerations for the adult females of the households, the majority were men because we did not have sufficient women enumerators. The observation was performed 24–48 h after the survey and all household members who were home at the time of the observation were observed. The adult female of the household, from whom consent was obtained, was told that observers would be noting the “daily routine” of the household and its members. No mention was made about handwashing, hygiene, or the survey. Observations were made in both morning and afternoon, with approximately 50 % of observations performed at each time.

Enumerators were trained to enter data directly into an electronic tablet using Open Data Kit (http://opendatakit.org/; University of Washington, Seattle, WA), an open-source platform for data entry.

This protocol was approved by the Ministry of Health of the Republic of South Sudan and the University at Buffalo Social and Behavioral Sciences Institutional Review Board.

### Analysis

Analysis was performed using SAS v9.3® (Cary, NC). Each camp was considered a stratum. Sampling weights were created based on the inverse probability of selection. For the analysis, stratification and sample weights were used to calculate weighted point estimates and sampling errors.

Structured observation analyses primarily evaluated handwashing behavior after toileting events and before food handling events.

Two wealth indices were created to examine the wealth or socioeconomic status (SES) of the survey households; one based on assets owned prior to relocation to Maban and a second for assets owned after relocating. Index values were generated for each participant and participants were assigned into quartiles.

Events observed during a structured observation within a household were not independent and, thus, household clustering was taken into account in the inferential statistics. SAS complex survey procedures with Taylor series linearization for variance estimation were used to take into account clustering, stratification, and unequal sample weights for both the survey and structured observation. Prevalence ratios were calculated using the Cox proportional hazards model for sample survey data via the SURVEYPHREG procedure in SAS. Significance tests were determined by chi-square test with p-value less than 0.05 considered as significant; 95 % confidence intervals were calculated.

## Results

### Demographics

We completed 195 interviews from the randomly selected list of the first 220 in Kaya. In Batil, the list of 230 was exhausted to yield 200. In Doro, we stopped after 205 interviews due to the civil unrest that erupted on December 15 2013 and the subsequent evacuation of several team members. Therefore, the total sample was 600 households with mean household size of seven with a mean of two children under five years old (range 0–9) per household. Of the 600 respondents, the median age was 28 years (range, 18–90 years). Of those respondents who could remember, nearly all (99 %) arrived in Maban in 2011 and 2012 and were thus present in the camps for at least 1 year. Households selected for structured observation were not different from the survey population with regard to household size, number of children under five years of age, soap and water access, or receipt of handwashing promotion messaging (Table 1).

**Table 1:** Demographic, access to hygiene materials, and messaging among participating women and household (HH) respondents, Maban County, 2013

Variables	All respondents Mean (median, range); (weighted)	Subset of HH receiving structured observation Mean (median, range); weighted
Demographics		
Age (years)	31.3 (28.0, 18–90)	NA
No. people in HH	7.0 (6.0, 1–31)	6.9 (6.0, 1–30)
No. children under 5 years in HH	1.8 (2.0, 0–9)	1.7 (2.0, 0–6)
	All Respondents	Structured Observation Subset
	% (n)	% (n)
Year arrived in Maban		
2011	58.5 % (351)	49.5 % (67)
2012	24.8 % (149)	30.9 % (35)
2013	0.5 % (3)	0
Don’t Know	16.2 % (97)	19.6 % (26)
Total	(600)	(128)
Access to materials needed for handwashing		
Frequency of water availability		
Always	21.3 % (128)	19.1 % (23)
Sometimes	69.7 % (418)	76.8 % (99)
Rarely	9.0 % (54)	4.1 % (6)
Self-reported time to walk to water source, collect water, and return		
Less than 5 min	45.5 % (273)	46.0 % (58)
5–30 min	38.8 % (233)	40.3 % (52)
Over 30 min	13.7 % (82)	11.9 % (15)
Presence of Soap in HH (observed)	97.2 % (575/596)	96.6 % (123/128)
Bought soap in last month	33.6 % (201/600)	37.4 % (48/128)
Number of HH sharing latrine		
<4	58.6 % (347)	61.4 % (75)
≥5	41.4 % (214)	38.6 % (39)
Total	(561)	(114)
Latrine had handwashing station		
Yes, overall	64.3 % (376)	74.6 % (88)
Soap and water at handwashing station		
Yes, overall	34.9 % (174)	---
Latrine had handwashing station		
1–4 HH sharing latrine-YES	69.8 % (252)	81.2 % (60)
≥5 HH sharing latrine-YES	58.4 % (118)	67.7 % (27)
Soap and water at handwashing station	54.2 % (174/376)	51.0 % (39/88)
1–4 HH sharing latrine-YES	53.7 % (113)	53.8 % (28)
≥5 HH sharing latrine-YES	57.5 % (60)	45.6 % (11)
Received hygiene messaging in past 3 months	84.5 % (514/600)	86.8 % (113/128)
Messaging talked about handwashing preventing disease	93.2 % (479/514)	92.3 % (104/128)

### Access to water, sanitation, soap and hygiene promotion

Walking to the water source, collecting water, and returning home was most often an endeavor of 30 min or less for the women of the household (Table 1). Most women (91 %) reported water was available “always” or “sometimes” at their preferred water source. Nearly all households (97 %) had bar soap available at the home at the time of the survey. A median of four households shared a communal latrine. Among all households, 64 % had a handwashing station near the latrine, yet only 35 % of all latrines had a functioning handwashing station with both water and soap present.

While 79 % of respondents expressed a preference to wash their hands near the latrine after defecation, only 59 % of those respondents had a handwashing station near the latrine and 60 % of those handwashing stations had soap present. Notably, of those who rinsed hands with water after toileting, 73 % of them *had* soap at the latrine handwashing station.

**Fig. 1:**
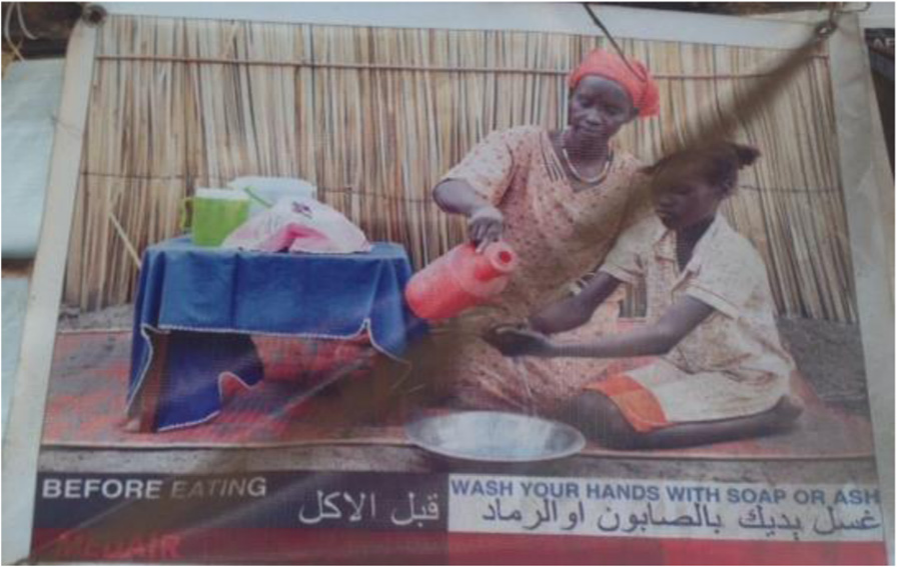
Handwashing promotion poster hung in Yusuf Batil Reception Center by Medair, a non-governmental organization

Eighty-five percent of women reported having heard messages promoting handwashing with soap, primarily from community health promoters, though posters were also present in reception centers for new arrivals (Fig. 1). Of these women, 93 % reported the message included information about handwashing preventing disease.

### Observed handwashing and associated factors

A total of 955 events were observed during which handwashing practices were assessed less than three days after the survey was conducted. These were primarily divided into toileting, 34 % of events, and “food handling” (preparing/cooking/eating), 38 % of events. Overall, 71 % of individuals were observed rinsing hands with water at these two critical times, 23 % were observed washing hands with soap, and 6 % of individuals were observed not washing at all.

**Fig. 2:**
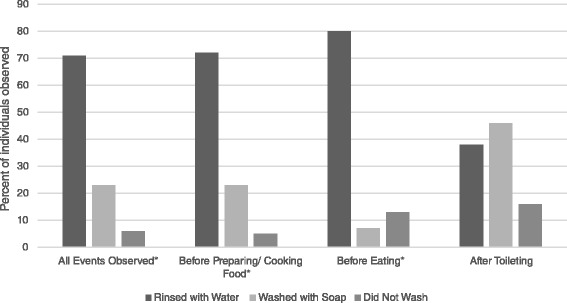
Observed household individuals hand cleansing behavior around critical times, percentage of individuals observed by event and the hand cleanesing behavior performed. *p < 0.001

Among household members observed “before food preparation/cooking”, 23 % washed hands with soap while an additional 72 % rinsed hands with water (Table 2). The discrepancy between reported and observed handwashing “before eating” was especially striking: 80 % of respondents self-reported that they washed hands with soap before eating, yet only 7 % were observed doing so (Fig. 2). Both “before food preparation/cooking” and “before eating” differences were statistically significant with a p-value < .001. When observed “after toileting”, 46 % of household members washed hands with soap, while 38 % rinsed with water and 16 % were not observed to wash at all.

**Table 2:** Weighted frequency of handwashing by knowledge, self-report, and observation among participating refugees, Maban County, 2013

Times for handwashing	A. What are the important times a person should use soap to wash hands?	B. In what situations do you use soap to wash your hands? (Self-report)	C. Proportion of events at which household members observed rinsing hands or washing hands with soap	D. Proportion of events at which household members observed washing hands with soap
	% (n; 95 % CI) *N* = 600	% (n; 95 % CI) *N* = 600	% (n/N; 95 % CI)	% (n/N; 95 % CI)
Before food prep/cooking	83.6 % (494; 80.7-86.7)	89.2 % (525; 86.7–91.6)	95.3 % (67/72; 89.5–100)	23.0 % (15/72; 10.1–36.0)
Before eating	78.9 % (483; 75.4–82.4)	80.3 % (480; 77.0–83.7)	86.5 % (204/248; 80.8–92.2)	6.5 % (20/248; 3.0–9.9)
Before feeding a child	12.2 % (67; 9.4–15.0)	15.4 % (87; 12.3–18.5)	94.2 % (37/40; 87.5–100)	26.8 % (11/40;12.2–41.3)
After cleaning child’s anus/ disposing feces	14.8 % (79; 11.7–17.9)	13.8 % (80; 10.9–16.7)	76.4 % (35/46; 64.0–88.9)	54.0 % (25/46; 38.2–69.7)
After toileting	73.6 % (441; 69.9–77.3)	74.3 % (446; 70.6–78.0)	83.6 % (219/263; 77.6–89.7)	45.6 % (111/263; 36.3–55.0)

Age was the only factor found to be associated with hand cleansing, including both rinsing and handwashing with soap—those over 15 years old performed more hand cleansing than those 15 years and younger. With 15 and under as the referent group, the prevalence ratio for washing hands with soap or rinsing hands after toileting for those aged over 15 years was 1.27 (*p* = 0.002). Similarly, compared to those under 15 years of age, the prevalence ratio for washing hands or rinsing before preparing, cooking, or eating food was 1.37 (*p* = <.0001) for those over 15 years of age.

No association was found between observed handwashing at critical times and sex of individual, location of handwashing, year of arrival to the camps, availability of soap, household size, number of children under 5 years in the household, whether messaging had been received, years of education for the respondent or her spouse, or wealth indices. The number of households that shared a latrine was not associated with the frequency of observed handwashing after toilet use. The presence of soap at the latrine handwashing station was also not associated with observed handwashing with soap after toileting.

The gap between knowledge and practice was evident when we examined the knowledge of when to wash hands with soap (Table 2, col A), self-reported handwashing with soap (Table 2, col. B) and the observed events of household members’ handwashing with soap or rinsing with water (Table 2, col. C/D). Knowledge and self-reported handwashing with soap was high for the critical times of “before food preparation/cooking”, “before eating”, and “after toileting”. Notable discrepancies emerged, however, when comparing the knowledge and self-reported handwashing behavior to the observed behavior. When rinsing with water and handwashing with soap were considered together, the rates of handwashing behavior were similar to the knowledge and self-reported practices; however, when handwashing with soap was considered alone, the rates decreased dramatically. It is notable that women seldom mentioned “before feeding a child” or “after cleaning a child” as times they wash hands with soap (15 % and 14 %, respectively); however upon observation, 27 % and 54 %, respectively were observed using soap. It appeared that handwashing with soap practices may be more diligent around childcare activities (Table 2).

## Discussion

In the humanitarian emergency in Maban, South Sudan, 1 year after a hepatitis E outbreak, rinsing hands with water was a frequently observed practice before eating, before feeding a child, preparing/cooking food and after toileting, but handwashing with soap was substantially less common. Despite increased soap, water, and latrine access, as well as intensified hand hygiene messaging in response to the 2012 Hepatitis E outbreak, handwashing with soap is not the norm after toilet use or before food handling for many refugees in Maban.

The observed frequencies of handwashing with soap were notably higher than those found in structured observation studies in developing country settings [5, 11]. Curtis et al. observed handwashing in 11 countries and found rinsing to be “in the order of three times more common than handwashing with soap” [5]. Rinsing with water does remove pathogens, but not as effectively as using soap [4]. Luby et al. described handwashing as a ladder: “handwashing with water is good; handwashing with soap is better” [12]. In this context, it appears most individuals do practice hand hygiene in the form of rinsing with water, but there remain barriers to soap use. Qualitative research would help elucidate these barriers. Our study design included a qualitative component but that part of our study was unable to be conducted due to the civil unrest which erupted in South Sudan during the last week of this survey. It could be that respondents prioritize soap for other chores, including laundry and bathing. It is also possible that given their reliance on outside organizations for provision of goods, refugees use their provisions sparingly so they are prepared for distribution delays or changes in good provided.

Although the knowledge of when to wash hands with soap was high, this did not translate into practice. In non-refugee settings, access to soap can also pose a barrier and improving soap access at the handwashing place has been found to double the likelihood of handwashing [13]. Water availability can also present a barrier in some settings [13]. Similarly, in non-emergency settings, the expense of using soap 10–20 times per day can be a barrier to using soap for handwashing [12]. We anticipated these barriers, yet these were not found to be associated with observed handwashing with soap practices in our study population. Most respondents continued to rinse with water alone, even when soap was widely available, distributed free each month, and present in nearly all households. Simply having soap is not enough to change handwashing behavior.

The exception to knowledge exceeding observed practice was among women caring for young children. Women were observed washing hands with soap more frequently than they reported doing so. The explanations are likely multifactorial but may be due in part to a habitualized behavior which women did not consciously consider when asked, “When are the important times to wash hands?” It could be a manifestation of nurturing to ensure they at least rinse their hands prior to feeding their child; or a matter of disgust motivating handwashing with soap after changing a diaper. It is notable that the percentage of both rinsing and handwashing was higher in practice than in self-report around these child care times, as we expected the self-reported rates to be higher than observed.

Information about the frequency of handwashing with soap was not available for the period prior to the HEV outbreak. Handwashing behavior observed in December 2013 may have represented an improvement over 2012 rates due to the intensive messaging; in the absence of behavioral information at baseline, we cannot know for certain whether there was an improvement in behavior resulting from the Hepatitis E prevention messaging. The fear of disease during the HEV outbreak may temporarily improve hand hygiene practices in response to an outbreak. [5] Over time, however, one study found individuals revert to their prior handwashing habits [5].

Clean water access is a cornerstone of emergency response and refugee camp management, and NGOs in Maban worked diligently to provide this during and after the hepatitis E outbreak. While handwashing with soap was also emphasized via posters and community health workers, more exploration into the behavioral determinants of handwashing behavior in this community is needed to design effective interventions that address not only gaps in knowledge, but also drivers and barriers to soap use. Unfortunately, we still do not know the best methods to support behavior change in emergency settings, and this warrants further investigation. Messaging is often based on a biomedical premise (i.e., germ theory), which does not take into account locally relevant beliefs. Didactic instructional messaging may be less effective than messages appealing to the emotive factors that can help shape behavior change, such as “disgust” and “comfort” [5]. Qualitative research to investigate beliefs, perceptions, motivators, and barriers among emergency-affected populations could help to optimize handwashing interventions. We had intended to conduct such research but due to the civil unrest that erupted in the Republic of South Sudan in December 2013, this was not possible.

### Limitations

The open-ended format of the self-report questions regarding times at which hands were washed with or without soap may have caused a lower response rate for some categories. That is, women were expected to name the times they wash hands with soap, rather than choose from a list, or be asked specifically about each critical time. This may account for the discrepancy between women who reported handwashing with soap before feeding a child and the women who were observed washing their hands. Another factor may be that the analysis was not limited to women with children under two years old as this information was not collected.

Structured observation is subject to the reactivity in which the circumstance of being observed may influence the participants’ behavior (Hawthorne effect) [10]. It is likely that, during structured observation, the participants may have washed hands with soap or rinsed more frequently than they would have if they had not been observed. On one hand, we assessed for reactivity by comparing the frequency of handwashing with soap during the first hour, compared to the second hour, and found no difference, suggesting no early reactivity that declined rapidly. On the other hand, it is possible that observed individuals were reactive throughout the entire duration of the observation.

Additionally, we observed all individuals of those present in the household at the time of the observation while the survey requested information on handwashing practices from a single individual; therefore, the low observed handwashing may not have correlated with the reported behavior. However, limiting the observations to women from the household (who were more likely to be the survey respondent) did not change the percentage of observed handwashing with soap events (data not presented). We were also not able to assess whether they washed their hands inside the latrine by bringing in an ibrik, however the small size of the latrine enclosure makes this less likely.

Due to logistical constraints, we stopped sampling when we reached 200 in Doro households and 195 in Kaya, which may have introduced some bias in that the sampling list of 250 and 230, respectively, were not completely sampled.

## Conclusion

The knowledge about when to use soap was high among the refugee population in Maban and a high proportion of individuals rinsed hands with water, which does confer some benefit [11]. A substantial gap existed between knowledge of when to wash hands with soap and the practice of using soap, despite nearly all households having soap available and receiving messaging on handwashing with soap. Given the high saturation of messaging, understanding of handwashing drivers through qualitative data collection and developing messages that address motivators and barriers to handwashing relevant to this population may be more effective than traditional health-focused messaging.

Refugee camps are a high risk setting for infectious diseases with epidemic potential. Diarrhea is a major cause of morbidity and mortality in emergencies besides the need to provide sufficient and safe water, there is a need to promote hand hygiene and provide soap that will be used for that activity [14]. Refugee camps provide an opportunity to optimize contextually relevant hand hygiene messaging and provide adequate WASH supplies and facilities. Both availability of materials and motivational messaging are needed to increase good hand hygiene practices and diminish disease among this vulnerable population.
